# Advances in CRISPR-Based Functional Genomics and Nucleic Acid Detection in Pigs

**DOI:** 10.3389/fgene.2022.891098

**Published:** 2022-05-31

**Authors:** Jinxue Ruan, Xuying Zhang, Shuhong Zhao, Shengsong Xie

**Affiliations:** ^1^ Key Laboratory of Agricultural Animal Genetics, Breeding and Reproduction of Ministry of Education & Key Lab of Swine Genetics and Breeding of Ministry of Agriculture and Rural Affairs, Huazhong Agricultural University, Wuhan, China; ^2^ Institute for Animal Breeding and Genetics, University of Veterinary Medicine Hannover, Hannover, Germany; ^3^ Hubei Hongshan Laboratory, Huazhong Agricultural University, Wuhan, China

**Keywords:** CRISPR, functional genomics, pig, nucleic acid detection, human biomedical model

## Introduction

The development of high-precision genome editing tools, such as targeted nucleases, has accelerated advances in fundamental human medicine, animal science, animal breeding, as well as disease diagnosis (Doudna and Charpentier, 2014; Kurtz et al., 2021; Rieblinger et al., 2021; Xie et al., 2021). In particular, the genome editing system known as CRISPR technology has grown rapidly since it was first reported (Jinek et al., 2012) and has become one of the most popular technologies. CRISPR/Cas9 technology can accurately identify target sequences and achieve efficient DNA cutting, thereby completing gene knock-outs/knock-ins on a genome-wide scale (Cong et al., 2013; Koike-Yusa et al., 2014).

However, due to double-strand breaks (DSBs) occurring during the editing process, this technology often introduces a large number of non-ideal InDel (insertion and deletion) mutations (Zhao et al., 2019). Subsequently, base editors (BEs), which can achieve precise editing of a single nucleotide using cytosine deaminase or adenosine deaminase without inducing DSB were developed (Gaudelli et al., 2017; Rees and Liu, 2018). Recently, prime editors (PEs) have further expanded the CRISPR-based-edit toolkit to all twelve possible base-to-base conversions, and insertion and deletion of short DNA fragments. This technology fuses reverse transcriptase and Cas9 protein, and uses a prime editing guide RNA (pegRNA) as the repair template to achieve precise gene editing (Anzalone et al., 2019). In this mini-review, we summarize and discuss recent applications of the CRISPR technology in pigs.

## Gene-Edited Pigs for Human Biomedicine

Pigs serve as an important agricultural resource and animal model in biomedical research. A variety of genetically modified pig models have been successfully generated through CRISPR-based technologies (Table 1) (Huang et al., 2020; Xu et al., 2020; Gu et al., 2021; Maeng et al., 2021; Yao et al., 2021; Yue et al., 2021; Xu et al., 2022). Duchenne muscular dystrophy (DMD) is an incurable X-linked inherited neuromuscular disorder and is caused by mutations in the dystrophin gene (*DMD*) (Hoffman et al., 1987). Studies in *mdx* (*X-linked muscular dystrophy*) mice, rats, dogs and monkey provided only a limited understanding of DMD disease mechanisms, as these possess different pathological manifestations from humans or cost highly (Nakamura et al., 2014; Chen et al., 2015; Nelson et al., 2016; Amoasii et al., 2018). Pigs (*Sus scrofa*) are closely related to humans in terms of anatomy, genetics and physiology. The generation of *DMD* knockout pig models using CRISPR/Cas9 technology may potentially pave the way for new treatments for patients (Yu et al., 2016; Zou et al., 2021).

**TABLE 1:** Summary of genetic changes introduced into porcine genome by CRISPR system.

Application	Gene symbol	Full name	Modification	Disease/Trait	References
Pig model for human biomedicine	DMD	Dystrophin	knock-out	Muscular Dystrophy	Yu et al. (2016)
					Zou et al. (2021)
	PPARγ	Peroxisome proliferator-activated receptor gamma	knock-in (MCK promoter-porcine PPARγ2 cDNA)	Oxidative fiber formation, intramuscular fat deposition	Gu et al. (2021)
	PBD-2	Porcine β-defensin 2	knock-in (PBD2-T2A-PBD2)	Anti-infection	Huang et al. (2020)
	MYF5, MYOD, MYF6	Myogenic Factor 5, myogenic differentiation 1	knock-out	Autologous muscle grafts	Maeng et al. (2021)
		myogenic Factor 6			
	MITF	Microphthalmia-associated transcription factor	Point mutation	Waardenburg syndrome 2A	Yao et al. (2021)
Agricultural production	CD163	Clusters of differentiation 163	knock-out	Porcine reproductive and respiratory syndrome virus and Transmissible gastroenteritis virus infection	Xu et al. (2020)
	ANPEP	Alanyl Aminopeptidase, Membrane			
	CD163	Clusters of differentiation 163	knock-out	Porcine reproductive and respiratory syndrome virus infection	Whitworth et al., (2016)
					Xu et al. (2020)
	CSN1S1	Casein Alpha S1	knock-in (porcine lactoferrin gene)	Survival rate of piglets	Han et al. (2020)
	ANPEP, CD163, MSTN, MC4R	Alanyl Aminopeptidase, Membrane	Targeted mutations	Economic traits	Wang X. et al. (2020)
		Clusters of differentiation 163, myostatin, melanocortin-4 receptor			
Identification of host factors restricting viral infection	EMC3, CALR	ER Membrane protein complex subunit 3	PigGeCKO library	Japanese encephalitis virus infection	Zhao et al. (2020)
		Calreticulin			
	TMEM41B	Transmembrane protein 41B	PigGeCKO library	Diverse viruses, Transmissible gastroenteritis virus, especially coronaviruses infection	Sun et al. (2021)
	ZDHHC17	Zinc finger DHHC-type palmitoyltransferase 17	Human (HeLa cells), GeCKO library screening	Swine acute diarrhea syndrome coronavirus	Luo et al. (2021)
	COG8	Golgi apparatus complex protein	GeCKO library screening	Influenza virus infection	Zhou et al. (2021)
	SMS1	Host sphingomyelin synthase 1	GeCKO library screening	Pseudorabies virus infection	Hölper et al. (2021)
	HBEGF	Heparin-binding EGF-like growth factor, diphthamide biosynthesis 1–5, Hsp40 member C24, Zinc Finger And BTB Domain Containing 17	GeCKO library screening	Diphtheria toxin	Yu et al. (2021)
	DPH1-5				
	DNAJC24				
	ZBTB17				
Xenotransplantation	GGTA1	Glycoprotein Alpha-Galactosyltransferase 1	knock-out	Immunological barriers	Butler et al. (2016)
					Petersen et al. (2016)
					Gao et al. (2017)
					Yue et al. (2021)
	CMAH	Cytidine monophospho-N-acetylneuraminic acid hydroxylase	knock-out	Immunological barriers	Butler et al. (2016)
					Yue et al. (2021)
					Gao et al. (2017)
	β4galNT2	β-1,4-N-acetyl-galactosaminyltransferase 2	knock-out	Immunological barriers	Yue et al. (2021)
	SLA class I	class I SLA molecules	knock-out	Immunological barriers	Reyes et al. (2014)
					Martens et al. (2017)
	iGb3S	Alpha 1,3-Galactosyltransferase 2	knock-out	Immunological barriers	Li et al. (2015)
	ULBP1	UL16 Binding protein 1	knock-out	Immunological barriers	Joanna et al. (2018)
	CIITA	Class II major histocompatibility complex transactivator	knock-out	Immunological barriers	Fu et al. (2020)
	B2M	Beta-2-Microglobulin	knock-out	Immunological barriers	Fu et al. (2020)
					Fischer et al. (2020)
	P53	Tumor protein P53	knock-out	Immunological barriers	Li H. et al. (2021)
	A3GALT2	Alpha 1,3-galactosyltransferase 2	knock-out	Immunological barriers	Shim et al. (2021)
	CD46	CD46 Molecule	Human gene knock-in (66 kb 5′ flanking region-CD46 gene-54 kb 3′ flanking region)	Immunological barriers	Fischer et al. (2016)
					Fischer et al. (2020)
					Yue et al. (2021)
	CD55	CD55 Molecule	Human gene knock-in (10 kb 5′ flanking sequence/1.8 kb CAG synthetic promoter- CD55 gene—6 kb 3′ flanking region)	Immunological barriers	Fischer et al. (2016)
					Fischer et al. (2020)
					Yue et al. (2021)
	CD59	CD59 Molecule	Human gene knock-in (10 kb 5′ flanking/promoter region-CD59 gene-37 kb 3′ flanking region)	Immunological barriers	Fischer et al. (2016)
					Fischer et al. (2020)
					Yue et al. (2021)
	CD47	CD47 Molecule	Human gene knock-in (PERVKO·3KO·9TG)	Immunological barriers	Yue et al. (2021)
	CD39	CD39 Molecule	Human gene knock-in (PERVKO·3KO·9TG)	Immunological barriers	Yue et al. (2021)
	HO1	Heme oxygenase-1	Human gene knock-in (SV40-driven hHO1 cDNA)	Immunological barriers	Fischer et al. (2016)
					Fischer et al. (2020)
	A20	TNF Alpha induced protein 3	Human gene knock-in (CAG-driven hA20 cDNA)	Immunological barriers	Fischer et al. (2016)
					Fischer et al. (2020)
	CD2	CD2 Molecule	Human gene knock-in (anti-CD2 mAb)	Immunological barriers	Nottle et al. (2017)
	B2M	Beta-2-Microglobulin	Human gene knock-in (PERVKO·3KO·9TG)	Immunological barriers	Yue et al. (2021)
	HLA-E	Major histocompatibility complex, class I, E	Human gene knock-in (PERVKO·3KO·9TG)	Immunological barriers	Yue et al. (2021)
	THBD	Thrombomodulin	Human gene knock-in (PERVKO·3KO·9TG)	Immunological barriers	Yue et al. (2021)
	EPCR	Endothelial cell protein C receptor	Human gene knock-in (0.7-kb hEPCR cDNA)	Immunological barriers	Lee et al. (2012)
	TFPI	Tissue factor pathway inhibitor	Human gene knock-in (PERVKO·3KO·9TG)	Immunological barriers	Yue et al. (2021)

## Gene-Edited Pigs for Agricultural Production

CRISPR technology offers a new strategy to combat infectious diseases in pigs. Porcine reproductive and respiratory syndrome (PRRS) is one of the most economically important swine infectious diseases worldwide. CD163 was identified as the striking receptor in PRRSV entry, and by knocking it out from the genome or editing the receptor using CRISPR/Cas9, pigs fully resistant to PRRSV have been produced - a milestone in modern pig breeding (Whitworth et al., 2016; Burkard et al., 2018; Xu et al., 2020). Another study reported the construction of genome-edited pigs with marker-free site-specific knock-in of *lactoferrin* gene in the 3′-end of *Casein alpha-s1* by CRISPR/Cas9-mediated homologous recombination (Han et al., 2020). Antibacterial activity of lactoferrin could potentially improve the survival rate of piglets in the genome-edited pigs (Han et al., 2020). There were abundant evidences that CRISPR-based technologies have great potential in human health and animal production.

## CRISPR-Based Functional Genomics to Combat Infectious Diseases

CRISPR technology provides an easy way to introduce targeted mutations into mammalian cells to induce loss-of-function phenotypes (Doudna and Charpentier, 2014; Hsu et al., 2014; Ruan et al., 2017). Genome-wide CRISPR screen has now been successfully applied to identify host factors that restrict viral infections, providing a powerful tool for exploring functional genomics of virus-host interactions (Shalem et al., 2014; Hoffmann et al., 2021). To identify novel host-dependent factors, a porcine genome-scale CRISPR/Cas9 knockout (PigGeCKO) library was established and successfully used to identify several key genes (*EMC3*, *CALR*, *etc.*) related to Japanese encephalitis virus (JEV) infection (Zhao et al., 2020). Several reports have identified multiple host factors required for the entry of other viruses and toxins in pigs and humans by using the CRISPR screening strategy (Hölper et al., 2021; Luo et al., 2021; Sun et al., 2021; Yu et al., 2021; Zhou et al., 2021).

Emerging coronaviruses (CoVs) pose a severe threat to human and animal health worldwide. Through CRISPR screening, transmembrane protein 41B (TMEM41B) was identified as a critical host-dependency factor required for the replication of diverse viruses, especially coronaviruses (Sun et al., 2021). TMEM41B was found to be involved in the formation of SARS-CoV-2 and transmissible gastroenteritis virus (TGEV) replicative organelles (Sun et al., 2021). ZDHHC17 (zinc finger DHHC-type palmitoyltransferase 17) was identified as a potential drug target for swine acute diarrhea syndrome coronavirus (SADS-CoV) infection by genome-wide CRISPR knockout library screening in human HeLa cells (Luo et al., 2021).

Adopting the same strategy, the Golgi apparatus complex protein (COG8) was identified as a pivotal regulator of influenza virus infection (Zhou et al., 2021). Host sphingomyelin synthase 1 (SMS1) was also found to be involved in pseudorabies virus (PRV) infection when the gD-mediated entry pathway was blocked (Hölper et al., 2021). In addition, HBEGF (heparin-binding EGF-like growth factor), DPH1-5 (diphthamide biosynthesis 1–5), DNAJC24 (Hsp40 member C24), and ZBTB17 were determined as diphtheria toxin (DT) receptors (Yu et al., 2021). These are the key factors involved in the biosynthesis of diphthamide, which serves as the molecular target for DT (Yu et al., 2021). These data demonstrate that CRISPR screening strategy is a powerful tool for functional genome in livestock.

Furthermore, CRISPR technology can also be used to specifically target infectious viruses (Freije and Sabeti, 2021). African swine fever (ASF) is a highly contagious viral disease of swine, with a high mortality rate up to 100%. CRISPR/Cas9 has been successfully used to produce recombinant ASF virus (ASFV), which could help speed up vaccine production to combat the infectious virus (Abkallo et al., 2021). Indeed, the CRISPR/Cas9 in combination with Cre/Lox system has been used to develop a stable anti-pseudorabies virus (PRV) vaccine of pig (Liang et al., 2016). Vaccination and challenge experiments demonstrate that recombinant vaccine candidates generated by gene editing technology can provide immune protection in pigs (Liang et al., 2016). These studies showed that development of virus vaccine can be accelerated via CRISPR and synthetic biology technologies.

## CRISPR-Based Diagnostics

The rapid detection of infectious diseases is highly needed in diagnosis and infection prevention (Pfaller, 2001; Hwang et al., 2018). CRISPR-based nucleic acid detection methods have suddenly emerged, with the potential to power the fields of genetic mutation and pathogen detection (Chen et al., 2018). This technology mainly employs Cas12, Cas13, and Cas14a, which have a target-activated trans-cleavage activity and can efficiently cleave single-stranded DNA (ssDNA) or single-stranded RNA (ssRNA) sequences (Gootenberg et al., 2017; Chen et al., 2018; Harrington et al., 2018).

To achieve point-of-care testing (POCT) of ASFV, a variety of sensitive diagnostic methods based on CRISPR technology have been established (He et al., 2020; Tao et al., 2020; Wang X. et al., 2020; Wu et al., 2020; Wei et al., 2022; Xie et al., 2022), for instance, recombinase-aided amplification (RAA)-Cas12a combined with lateral flow detection assay (Wang Y. et al., 2020), CRISPR/Cas12a based universal lateral flow biosensor assay (Wu et al., 2020), CRISPR/Cas12a enhanced fluorescence assay (Tao et al., 2020), CRISPR/Cas13 combined with lateral flow strip assay (Wei et al., 2022), as well as high-throughput and all-solution phase ASFV detection assay (He et al., 2020). Recently, to simplify the detection process, the rapid visual CRISPR assay (RAVI-CRISPR), combining a naked-eye colorimetric detection method based on CRISPR/Cas12a and a convolutional neural network was established (Xie et al., 2022). This RAVI-CRISPR/MagicEye mobile APP system is perhaps the today’s simplest platform for rapid POCT testing.

## Porcine Genome Engineering for Xenotransplantation

The extreme shortage of human donor organs for the treatment of patients with end-stage organ failure is well known. Pig-to-human xenotransplantation is a most promising strategy to solve this problem, because domestic pigs are similar to humans in terms of anatomy, physiology and organ size, and are highly reproductive and low in maintenance costs (Hryhorowicz et al., 2017). However, discrepancies between pigs and humans lead to the development of immune barriers, blocking direct xenotransplantation (Vadori and Cozzi, 2015).

In the last decade, CRISPR technology accelerated the pace and extent of modifications to porcine genomes, such as knockout of major carbohydrate antigens (*GGTA1*, *CMAH*, *β4galNT2*) and tumor suppressor protein (*p53*), as well as knockin of various human complement regulatory proteins (e.g. *CD46*, *CD55*), human coagulation regulatory proteins (e.g. *THBD*, *EPCR*), human anti-inflammatory molecule (*HO1*), and human macrophage-inhibitory ligand (*CD47*), to modulate human immune response (Cooper et al., 2019; Li H. et al., 2021). These genetically modified pigs have been used in preclinical studies and greatly improved survival outcomes of xenografts of non-human primate recipients (Niu et al., 2021). In addition, multiplex CRISPR/Cas9 gene editing technology has enabled multi-fold knockouts of porcine genes in various combinations. Pigs carrying multi-fold xenoprotective transgenes and knockouts of xenoreactive antigens have been generated (Fischer et al., 2016; Zhang et al., 2018; Fischer et al., 2020; Fu et al., 2020; Shim et al., 2021; Yue et al., 2021), with great potential to completely eliminate immunological barriers. It remains a challenge, however, to effectively assess the human immune response induced by various genetic modifications and to identify the ideal gene combinations (Li P. et al., 2021). Recently, the world’s first porcine-to-human transplantation was performed at the University of Maryland Medical Center, successfully transplanting a genetically modified porcine heart into a 57-year-old man with end-stage heart disease, and the patient lived for two months after the transplant (Shah and Han, 2022). The advent of the CRISPR system has accelerated the field, bringing the successful application of xenotransplantation closer to reality.

## Conclusion and Regulation of CRISPR Development

**FIGURE 1:**
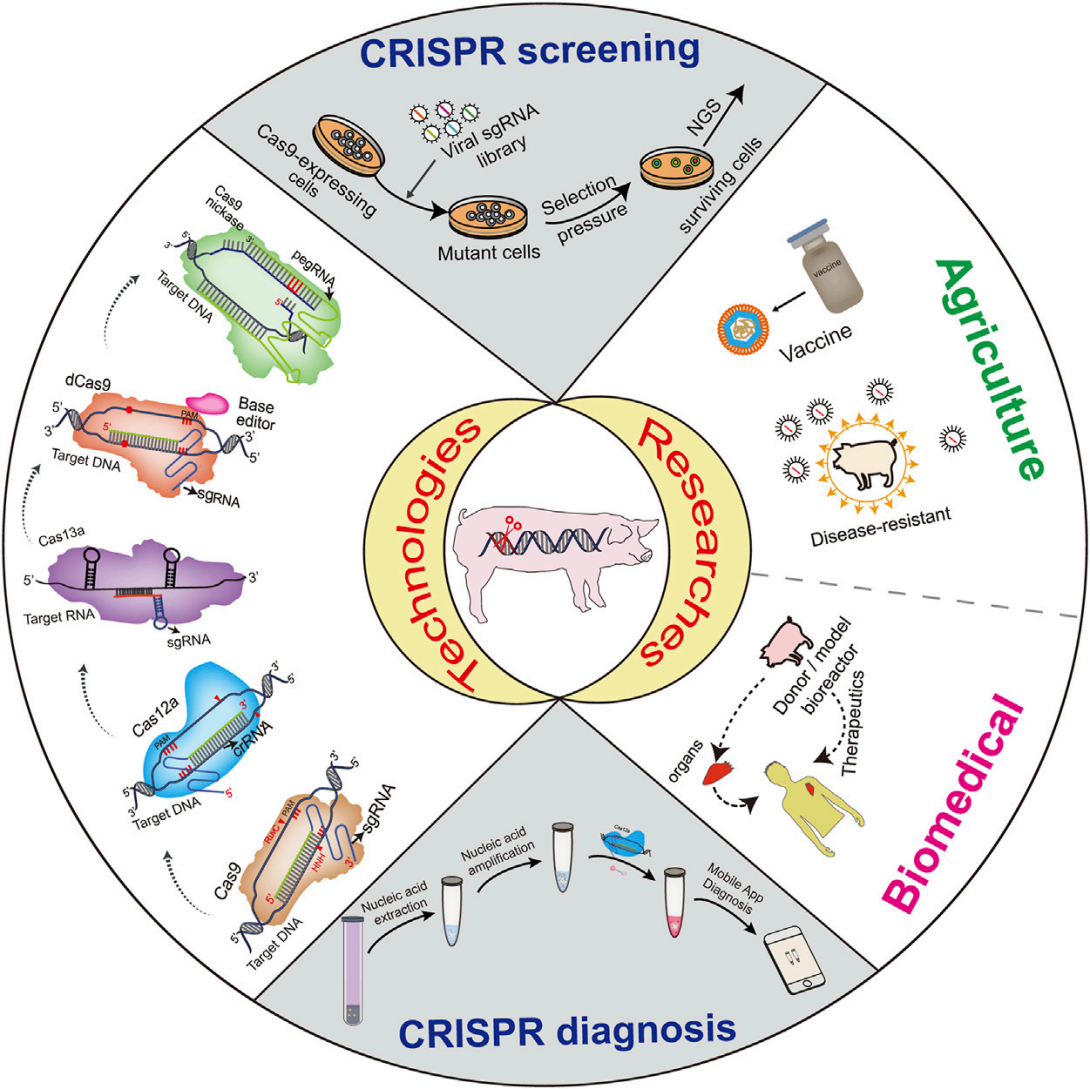
The CRISPR system and its applications.

CRISPR, a sequence-specific nuclease able to edit target gene sequences, has ignited a revolution in the field of genetic engineering and site-specific editing within malfunctioning genes (Hsu et al., 2014). The system’s efficiency, robustness, and affordability allow its application to endless potential genetic targets (Figure 1). The use of CRISPR in genetic disorders, infectious diseases, defective traits and immunological barriers via gene knockout, gene knockin and gene editing has immense potential for the development of animal production, human medicine and Xenotransplantation (Doudna and Charpentier, 2014; Hsu et al., 2014; Ruan et al., 2017; Shah and Han, 2022). CRISPR technology has also been extensively employed to develop rapid point-of-care detection methods for viruses (Xie et al., 2022), with great potential in combating infectious diseases such as CoVs and ASFV. Additionally, the technology exerts important roles in clarifying the pathways of virus-host interactions, and generating recombinant viruses to speed up vaccine production. Future applications of CRISPR will enhance the quality and quantity of gene therapy and animal production, improve human health and animal welfare and will save countless lives.

Gene editing regulations for animals have not yet been globally established and vary greatly between countries. China’s regulations on genetically modified organisms (“GMOs”) mainly focus on Agricultural GMOs. In the U.S., genetically modified crops are regulated by the U.S. Department of Agriculture, which is relaxing its oversight of gene editing. While animal biotechnology is regulated by the Food and Drug Administration (FDA) under an unusual reading of the Federal Food, Drug, and Cosmetic Act of 1938, and gene editing is very strictly regulated by the FDA. In our opinion, using CRISPR technology, we can create an advanced animal that is essentially identical to the original one in all respects. Nevertheless, it is important to establish sound laws and regulations on CRISPR in the worldwide scientific community and between government agencies globally. Despite all risks, we believe that the application of CRISPR will provide benefits for everyone in the not far-distant future.

## Insights and Prospects

The rapid development of life science has brought us from the “reading” stage of biological genetic information to the post-genome era, in which “rewriting” and even “new design” of genomes are gradually becoming a reality. Synthetic biology, which aims to design and create new living organisms, has developed rapidly under this background and has shown great promise for applications in biomedicine, agriculture, vaccines, manufacturing, and energy. In continuous exploration and research, gene editing technologies, especially CRISPR, have evolved from initial reliance on naturally occurring homologous recombination in cells to targeted cleavage at almost any site, and even to nucleic acid-based diagnostics. The simplicity and efficiency of its operation has greatly facilitated the genetic modification of species and disease diagnosis. Gene editing provides the means for continued modification of synthetic life and opens up more possibilities for the creation of new species through genetic modification. De novo genome synthesis and the large-scale modifications of natural genomes belong to the fields of synthetic genomics and gene editing (Xie et al., 2017), both subjects are current hot spots topics in synthetic biology research.

Since Science magazine named CRISPR technology the breakthrough of the year in 2015, this new technology has taken the gene-editing field by storm. In the past few years, CRISPR technology has rapidly swept the animal world as a popular gene editing technique. Although the research and application of gene editing technology has been developing rapidly, gene editing technology still faces challenges in terms of off-target, ethics and safety. The future development of gene editing technology needs to pay attention to the following aspects: first, strengthen planning and guidance, and attach great importance to strengthening research on basic theories and innovative methods of gene editing; second, strengthen supervision and scientific guidance, and pay attention to the applications of gene editing; third, strengthen research on ethical norms, improve the legal and policy system for gene editing supervision, and vigorously support the research and development of animal gene editing products; fourth, strengthen the popularization of science, let more people understand and accept gene editing technology, so that gene editing can better benefit mankind.
